# Small Antimicrobial Resistance Plasmids in Livestock-Associated Methicillin-Resistant *Staphylococcus aureus* CC398

**DOI:** 10.3389/fmicb.2018.02063

**Published:** 2018-09-19

**Authors:** Andrea Feßler, Kristina Kadlec, Yang Wang, Wan-Jiang Zhang, Congming Wu, Jianzhong Shen, Stefan Schwarz

**Affiliations:** ^1^Institute of Microbiology and Epizootics, Centre for Infection Medicine, Department of Veterinary Medicine, Freie Universität Berlin, Berlin, Germany; ^2^Institute of Farm Animal Genetics, Friedrich-Loeffler-Institut, Neustadt, Germany; ^3^Beijing Advanced Innovation Center for Food Nutrition and Human Health, College of Veterinary Medicine, China Agricultural University, Beijing, China; ^4^State Key Laboratory of Veterinary Biotechnology, Harbin Veterinary Research Institute, Chinese Academy of Agricultural Sciences, Harbin, China

**Keywords:** LA-MRSA, *erm*, *lnu*(A), *cfr*, *vga*, *spd*, *apmA*, *dfrK*

## Abstract

Livestock-associated methicillin-resistant *Staphylococcus aureus* (LA-MRSA) isolates of the clonal complex 398 are often resistant to a number of antimicrobial agents. Studies on the genetic basis of antimicrobial resistance in these bacteria identified SCC*mec* cassettes, various transposons and plasmids of different sizes that harbor antimicrobial resistance genes. While large plasmids that carry multiple antimicrobial resistance genes – occasionally together with heavy metal resistance genes and/or virulence genes – are frequently seen in LA-MRSA ST398, certain resistance genes are also associated with small plasmids of up to 15 kb in size. These small resistance plasmids usually carry only one, but in rare cases also two or three antimicrobial resistance genes. In the current review, we focus on small plasmids that carry the macrolide-lincosamide-streptogramin B resistance genes *erm*(C) or *erm*(T), the lincosamide resistance gene *lnu*(A), the pleuromutilin-lincosamide-streptogramin A resistance genes *vga*(A) or *vga*(C), the spectinomycin resistance gene *spd*, the apramycin resistance gene *apmA*, or the trimethoprim resistance gene *dfrK*. The detailed analysis of the structure of these plasmids allows comparisons with similar plasmids found in other staphylococci and underlines in many cases an exchange of such plasmids between LA-MRSA ST398 and other staphylococci including also coagulase-negative staphylococci.

## Introduction

Isolates of the clonal complex 398 (CC398) are the most frequently encountered livestock-associated methicillin-resistant *Staphylococcus aureus* (LA-MRSA) in Europe as well as Northern America (Cuny et al., 2013). It is assumed to have developed from methicillin-susceptible *S. aureus* (MSSA), which has gained methicillin and tetracycline resistance after its introduction into animal hosts (Price et al., 2012). In contrast, LA-MRSA ST9 is the most widespread and most important LA-MRSA type in China (Li et al., 2017).

Many antimicrobial resistance genes in staphylococci of human and animal origin are located on plasmids (Wendlandt et al., 2013a; Schwarz et al., 2014). These include original plasmid-borne resistance genes, but also transposon-borne resistance genes in cases when the corresponding transposon has integrated into a plasmid or recombination between a resistance gene-carrying transposon and a plasmid has occurred. Plasmids play an important role in the dissemination of antimicrobial resistance genes among staphylococci (Schwarz et al., 2014). In this regard, LA-MRSA isolates do not differ from other staphylococci. Previous studies showed that LA-MRSA of CC398 can act as a donor and as a recipient in the dissemination of antimicrobial resistance plasmids, and thereby plays an important role in the mobilome of firmicutes (Schwarz et al., 2014). Over the years, several novel or unusual resistance genes have been found in LA-MRSA CC398 (Kadlec et al., 2012b).

In this review, we describe selected small antimicrobial resistance plasmids (<15 kb in size) that have been identified in LA-MRSA of CC398 and their relationships to similar plasmids of other staphylococcal species (**Table 1**).

**Table 1:** Small antimicrobial resistance plasmids in livestock-associated MRSA/MSSA CC398^∗^.

Plasmid	Size (bp)	Rep family^∗∗^	Origin	Antimicrobial resistance genes	Reference	Accession number(s)
pSWS371	2458	Rep_1	Chicken house	*erm*(C)	Wendlandt et al., 2014b	NC_024963.1; HG380317.1
pSWS372	3982	Rep_2	Chicken house	*erm*(C)	Wendlandt et al., 2014b	NC_024964.1; HG380318.1
pUR3912	6176	Rep_1	Human	*erm*(T)	Gómez-Sanz et al., 2013a	HE805623
pLNU1	2361	Rep_1	Pig	*lnu*(A)	Lozano et al., 2012b	NZ_AVBD01000030.1
pCPS32	5718	Rep_trans	Dust sample pig farm	*vga*(A)	Kadlec et al., 2010	NC_019141.1
pVGA	5713	Rep_trans	Human	*vga*(A)	Lozano et al., 2012a	FJ207465.1; NC_011605.1
pUR2355	7609	Rep_L	Human	*vga*(A)	Lozano et al., 2012a	JQ312422.1; NC_019145.1
pUR4128	7567	Rep_L	Pig	*vga*(A),	Lozano et al., 2012a	JQ861960.1; NC_019147.1
pKKS825	14365	PriCT_1 + REP_3 + Rep_1	Pig	*vga*(C), *aadD*, *dfrK, tet*(L)	Kadlec and Schwarz, 2009b	NC_013034.2; FN377602.2
pCPS49	5292	Rep_2	Dust sample pig farm	*vga*(C)	Kadlec et al., 2010	NC_019142.1; FN806792.1
pDJ91S	3928	Rep_trans	Chicken	*spd*	Jamrozy et al., 2014	KC895984.1
pSWS2889	3898	Rep_trans	Human	*spd*	Wendlandt et al., 2014a	NC_023385.1; HG803547.1
pKKS49	4809	untypeable	Dust sample pig farm	*apmA*	Kadlec et al., 2012a	NC_019149.1; HE611647.1
pKKS627	6243	untypeable	Pig	*dfrK*; *tet*(L)	Kadlec and Schwarz, unpublished	NC_014156.1; FN390948.1

*^∗^All plasmids originate from MRSA isolates except plasmid pUR3912, which originates from a MSSA isolate.*

*^∗∗^The classification of the plasmids into Rep families follows the information published in Lanza et al. (2015).*

## Small Plasmids Carrying *erm*(C) Genes

**FIGURE 1:**
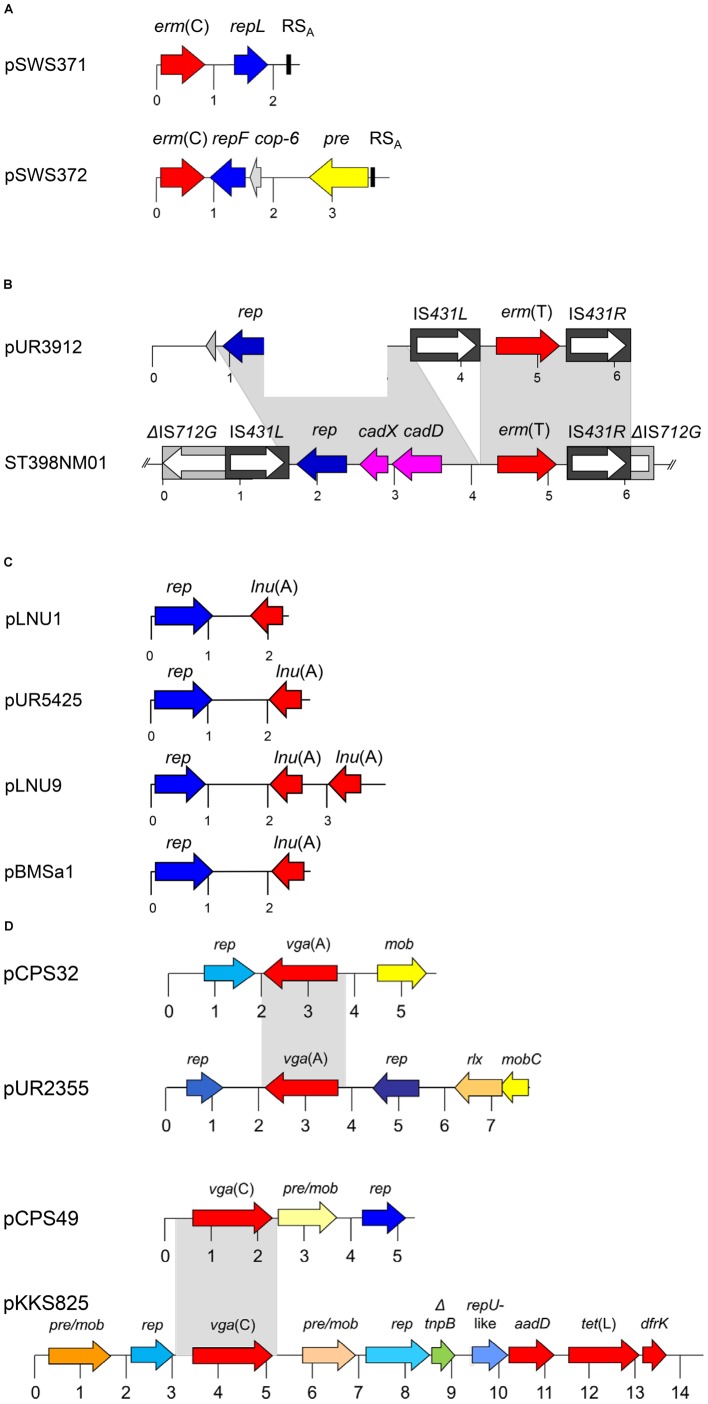
Schematic presentation of the organization of **(A)** the two *erm* (C)-carrying plasmids pSWS371 and pSWS372, **(B)** the *erm*(T)-carrying plasmid pUR3912 and the chromosomal *erm*(T) region of MRSA ST398 isolate ST398NM01, **(C)** the *lnu*(A)-carrying plasmids pLNU1, pLNU9, pUR5425, and pBMSa1, and **(D)** the *vga*(A)- and the *vga*(C)-carrying plasmids pCPS32, pUR2355, pCPS49 and pKKS825 from MRSA ST398. The reading frames are presented as arrows with the arrowhead indicating the direction of transcription. A distance scale in kb is given below the maps. The resistance genes are indicated in red, plasmid replication genes *rep* in different shades of blue, and genes involved in plasmid recombination, mobilization and relaxation *pre*, *mob*, *pre/mob*, and *rlx* in different shades of yellow/-orange. The different blue and yellow-orange shadings indicate differences in the respective genes. In panel **(A)**, the plasmid copy control gene *cop-6* is displayed in gray and the staphylococcal recombination site A (RS_A_) is indicated by a black box. In panel **(B)**, the cadmium resistance operon *cadDX* is shown in pink and the IS*431* elements and the IS*712*G element are displayed as black or gray boxes, respectively, with the white arrow inside representing the transposase gene *tnp*. The gray-shaded areas represent areas of at least > 95% sequence identity. This figure is modified from Lüthje et al. (2007) and Kadlec et al. (2010), Gómez-Sanz et al. (2013a), and Wendlandt et al. (2014b).

The gene *erm*(C) is the most widespread *erm* gene among staphylococci (Schwarz et al., 2014; Feßler et al., 2018). It is mainly located on plasmids. The *erm*(C) gene codes for a rRNA methyltransferase that targets the adenine residue at position 2048 in 23S rRNA and confers resistance to macrolides, lincosamides, and streptogramin B (MLS_B_) antibiotics. Its expression can be inducible or constitutive, based on the completeness of the translational attenuator that is located upstream of the *erm*(C) gene. So far, three types of small plasmids that carry solely the *erm*(C) gene have been identified in staphylococci (Schwarz et al., 2014; Feßler et al., 2018) – two of them also among LA-MRSA from a chicken house environment (Wendlandt et al., 2014b; **Figure 1A** and **Table 1**).

Two small *erm*(C)-carrying plasmids, pSWS371 and pSWS372, were identified in the same LA-MRSA CC398 isolate of *dru* type dt11 and *spa* type t3015. Plasmid pSWS371 has a size of 2458 bp and harbored – besides the *erm*(C) gene – only the plasmid replication gene *repL*. Plasmid pSWS372 has a size of 3982 bp and carried the plasmid replication gene *repF*, a new type of plasmid recombination and mobilization gene *pre*/*mob*, and the *cop-6* gene possibly involved in the control of the copy number of this plasmid. The expression of both *erm*(C) genes was constitutive as explained by 16-bp (pSWS371) and 74-bp (pSWS372) deletions in the respective *erm*(C)-associated translational attenuators. After separate transformation of each plasmid into *S. aureus* RN4220, both plasmids were functionally active and conferred the expected MLS_B_ resistance phenotype. The observation that both *erm*(C)-carrying plasmids stably coexist in the same bacterium may be explained by the fact that they belong to different incompatibility groups because they belong to different plasmid replication families (Wendlandt et al., 2014b).

Plasmid pSWS371 resembles in its structure and nucleotide sequence a number of small *erm*(C)-carrying plasmids which have been identified not only in *S. aureus*, but also in various coagulase-negative staphylococci from humans and animals (Schwarz et al., 2014; Feßler et al., 2018), including the prototype plasmid pNE131 from human *Staphylococcus epidermidis* (Lampson and Parisi, 1986). In contrast, plasmid pSWS372 is closely related to the prototype plasmid pE194 from human *S. aureus* (Horinouchi and Weisblum, 1982). Plasmids related to pE194 have so far only rarely been detected among staphylococci from humans and animals (Lodder et al., 1996; Schwarz et al., 2014).

It should be noted that small *erm*(C)-encoding plasmids can be integrated in part or completely into larger plasmids. In the case of the approximately 25-kb *erm*(T)-carrying plasmid pUR2940 of human LA-MRSA CC398, a complete 2363-bp *erm*(C)-carrying plasmid was integrated via insertion sequences of the type IS*Sau10* (Gómez-Sanz et al., 2013b). Moreover, the 7057-bp plasmid pSS-03 identified in various CoNS species of pigs in China carried an *erm*(C) gene together with the multidrug resistance gene *cfr* (Wang et al., 2011). The closely related 7054-bp plasmid pMSA16, in which, the *erm*(C) gene was replaced by a Tn*554*-analogous *erm*(A) gene, was identified in a LA-MRSA ST9 of bovine origin in China (Wang et al., 2012). Most recently, another related plasmid, the 8558-bp plasmid pSEM13-0451, which carried the genes *erm*(T) and *cfr* was detected in methicillin-resistant *S. epidermidis* of human origin (Lazaris et al., 2017).

## Small Plasmids Carrying *erm*(T) Genes

Like *erm*(C), the gene *erm*(T) also confers inducible or constitutive MLS_B_ resistance and is also preceded by a translational attenuator. The *erm*(T) gene has been found in LA-MRSA and MSSA of ST398 and can be located either in the chromosomal DNA or on plasmids of different sizes (Kadlec and Schwarz, 2010a; Vandendriessche et al., 2011; Gómez-Sanz et al., 2013a,b).

The *erm*(T) gene was first described in staphylococci on the approximately 40-kb plasmid pKKS25 from a porcine LA-MRSA ST398 in Germany. In this plasmid, the *erm*(T) gene was found together with the trimethoprim resistance gene *dfrK* and the tetracycline resistance gene *tet*(L) on an approximately 4.6 kb segment that was flanked by IS*Sau10* elements in the same orientation (Kadlec and Schwarz, 2010a). A smaller plasmid, the 6176-bp plasmid pUR3912, was isolated from a human MSSA ST398-t571 isolate in 2011 in Spain (**Table 1**). This plasmid harbors the *erm*(T) gene flanked by two IS elements in the same orientation, a plasmid replication gene *rep* and a functionally *cadDX* operon for cadmium resistance (Gómez-Sanz et al., 2013a). This plasmid showed striking homology to a chromosomal segment found in the MSSA strain ST398NM01 (Uhlemann et al., 2012) (**Figure 1B**). In a different study, the original strain carrying plasmid pUR3912 was analyzed in more detail and a chromosomal copy of plasmid pUR3912 was found in addition to the extrachromosomal location (Gómez-Sanz et al., 2013c). Plasmid pUR3912 was able to integrate into and excise from the chromosome of the corresponding MSSA isolate via the IS elements (Gómez-Sanz et al., 2013c). The closely related insertion sequences IS*257*, IS*431*, and IS*Sau10*, play an important role in the integration of small resistance plasmids into the chromosomal DNA or into other plasmids (Schwarz et al., 2014).

## Small Plasmids Carrying *lnu*(A) Genes

The gene *lnu*(A) codes for a lincosamide nucleotidyltransferase that confers solely low-level lincosamide resistance. The first complete sequence of an *lnu*(A)-carrying plasmid was from a bovine *S. aureus* isolate from Mexico. The corresponding plasmid pBMSa1 had a size of 2750 bp and carried only the *lnu*(A) gene and a plasmid replication gene *rep* (Loeza-Lara et al., 2004). In later studies on CoNS from bovine mastitis cases in Germany, nine novel types of *lnu*(A)-carrying small plasmids – pLNU1 to pLNU9 – have been identified (Lüthje et al., 2007). Plasmids pLNU1 to pLNU9 were similar to each other and to pBMSa1 in their structures and in their organization. They varied in size between 2278 bp and 3783 bp (Lüthje et al., 2007). In a study on MRSA and other staphylococci of human and animal origin conducted in Spain, Lozano and co-workers found a plasmid identical to pLNU1 (**Figure 1C**) in a porcine LA-MRSA ST398-t108 isolate (**Table 1**) and in a porcine methicillin-resistant *Staphylococcus sciuri* isolate. Moreover, they identified a novel type of *lnu*(A)-carrying plasmid, the 2690-bp plasmid pUR5425, which was next related to plasmid pLNU4, in a human MRSA ST125-t067 isolate (Lozano et al., 2012b).

## Small Plasmids Carrying *vga*(A) Genes

The gene *vga*(A) codes for an ABC-F protein that mediates resistance by protecting the ribosome against lincosamides, pleuromutilins, and streptogramin A antibiotics (Sharkey et al., 2016). Among the various *vga* genes so far identified in staphylococci, the *vga*(A) genes are most widespread. They may be located either on plasmids of variable sizes or on transposon Tn*5406* (Haroche et al., 2002). The *vga*(A) genes have also been found on small plasmids, including the 5713-bp plasmid pVGA from a human *S. aureus* of unknown MLST type in Portugal (Gentry et al., 2008) and the closely related 5718-bp plasmid pCPS32 from an LA-MRSA ST398 which originated from a dust sample taken at a swine farm in Portugal (Kadlec et al., 2010; **Figure 1D** and **Table 1**). These plasmids harbor three genes: the *vga*(A) gene, a plasmid mobilization gene *mob*, and a plasmid replication gene *rep*. In a study from Spain, Lozano et al. (2012a) detected plasmid pVGA in two LA-MRSA ST398 from humans. In addition, they identified four novel *vga*(A)-carrying plasmids among LA-MRSA ST398 from humans and pigs, but also in a human methicillin-resistant *S. epidermidis* ST83, a feline methicillin-resistant *S. epidermidis* ST60, and human methicillin-susceptible *S. epidermidis* ST100 (Lozano et al., 2012a). These four novel plasmids ranged in size between 7209 and 7609 bp. The two larger plasmids pUR4128 and pUR2355 had a similar structure which comprised – in addition to the *vga*(A) gene – a mobilization gene *mobC*, a relaxase gene *rlx*, two plasmid replication genes *rep*, and a small ORF of unknown function. The two smaller plasmids pUR3937 and pUR3036 carried – besides the *vga*(A) gene – a *rlx* gene, three *mob* genes, a single *rep* gene, and three small ORFs of unknown function (Lozano et al., 2012a). In a study from China on dogs and cats and their owners, Deng et al. (2017) found only 13 staphylococcal isolates with elevated pleuromutilin MICs. One of them, a human *S. epidermidis* isolate, harbored the *vga*(A) gene on plasmid p132R (7209 bp) which shared 99% nucleotide sequence identity with the same-sized plasmid pUR3036 of feline origin (Lozano et al., 2012a). Another small *vga*(A)-carrying plasmid, pSWS581 (6311 bp) was identified in a bovine *S. epidermidis* isolate from Germany (Wendlandt et al., 2015a).

A variant of the gene *vga*(A), designated *vga*(A)_LC_, was found on plasmids p131R and p131A, both 6056 bp in size and originating from *S. haemolyticus* isolates of human and feline origin in China (Deng et al., 2017). Both plasmids differed only by 9 bp from each other and shared 99% nucleotide sequence identity with plasmid pUR2355 from a human *S. aureus* ST398-t011 isolate in Spain (Deng et al., 2017).

## Small Plasmids Carrying *vga*(C) Genes

The *vga*(C) gene was described in a LA-MRSA isolate from pig origin in Germany. In this isolate, the *vga*(C) gene was located on the plasmid pKKS825 (Kadlec and Schwarz, 2009b). This plasmid has a size of 14365 bp. Besides the *vga*(C) gene that confers resistance to pleuromutilins, lincosamides, and streptogramin A antibiotics, plasmid pKKS825 harbored the gene *aadD* for kanamycin/neomycin resistance, the gene *dfrK* for trimethoprim resistance, and the gene *tet*(L) for tetracycline resistance. In addition, plasmid pKKS825 carried two *pre*/*mob* genes and three *rep* genes (Kadlec and Schwarz, 2009b). The *vga*(C) gene was also found on smaller plasmids, such as the 5292-bp plasmid pCPS49 which originated from a dust sample taken in a breeding pig farm in Portugal (Kadlec et al., 2010; **Figure 1D** and **Table 1**). This plasmid harbored a *rep* gene and a *pre*/*mob* gene which were unrelated to the corresponding genes usually present on staphylococcal plasmids. Based on the analysis of the *rep* and *pre*/*mob* genes, it is assumed that plasmid pCPS49 may have developed in bacteria other than staphylococci. Sequence homology between plasmids pCPS49 and pKKS825 was limited to the *vga*(C) gene and 404 bp in the upstream and 249 bp in the downstream region (Kadlec et al., 2010).

## Small Plasmids Carrying *spd* Genes

The gene *spd* codes for a spectinomycin adenyltransferase and is one of the three so far known spectinomycin resistance genes in staphylococci (Murphy, 1985; Wendlandt et al., 2013b, 2014c; Jamrozy et al., 2014). The gene *spd* is usually located on small plasmids of <5 kb in size (Jamrozy et al., 2014; Wendlandt et al., 2014a, 2015b).

**FIGURE 2:**
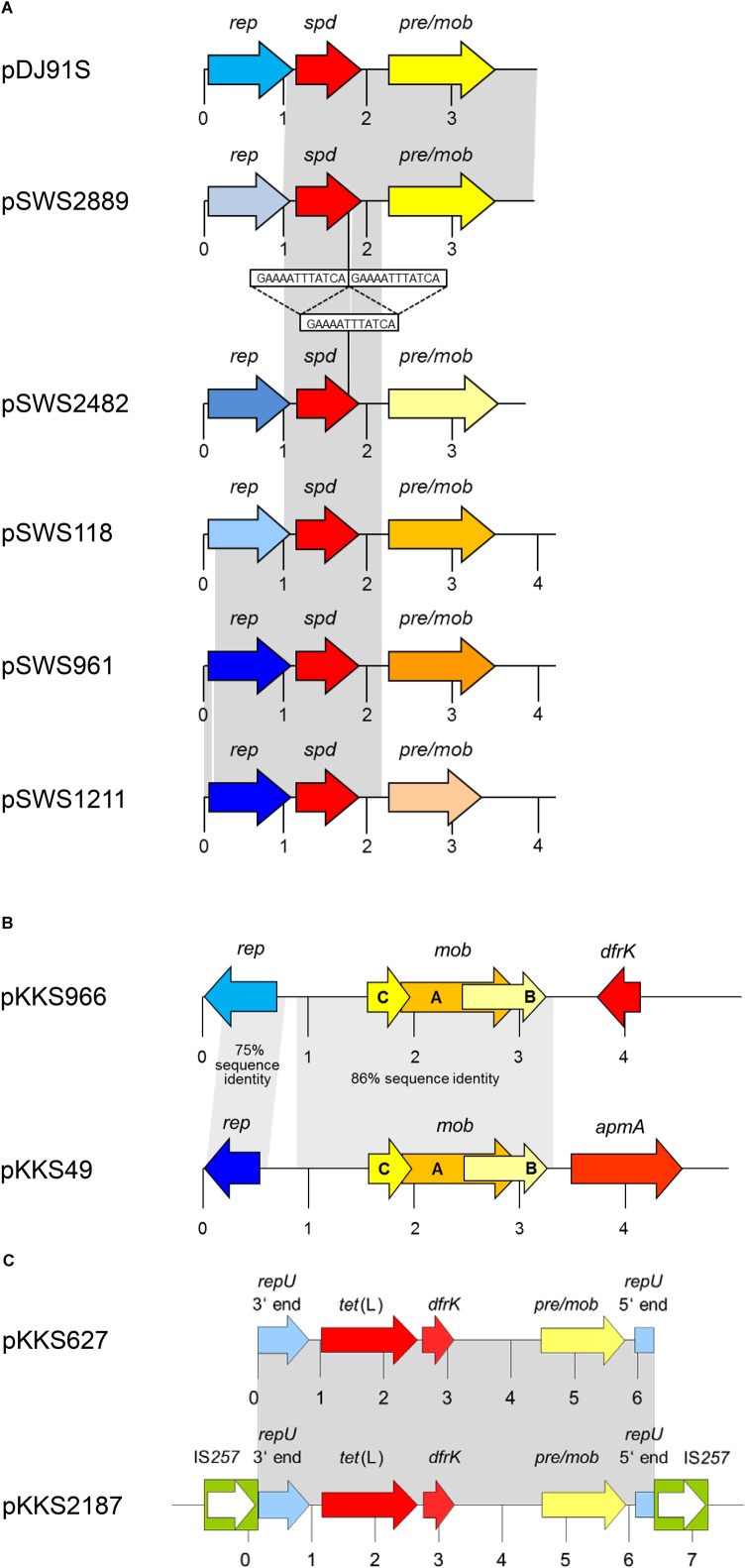
Schematic presentation of **(A)** the *spd*-carrying plasmids pDJ91S and pSWS2889 from MRSA ST398 in comparison to closely related plasmids pSWS2482 and pSWS1211 from *S. hyicus*, pSWS118 from *S. chromogenes*, and pSWS961 from *S. equorum*, **(B)** the *apmA*-carrying plasmid pKKS49 from MRSA ST398 and the *dfrK*-carrying plasmid pKKS966 from *S. hyicus*, and **(C)** the *dfrK*-carrying plasmid pKKS627 and the *dfrK* region of plasmid pKKS2187, both from MRSA ST398. The reading frames are presented as arrows with the arrowhead indicating the direction of transcription. A distance scale in kb is given below the maps. The resistance genes are indicated in red, plasmid replication genes *rep* in different shades of blue, and genes involved in plasmid recombination and mobilization, *pre/mob* and *mob*, in different shades of yellow/-orange. The different blue and yellow-orange shadings indicate differences in the respective genes. The IS*257* elements in panel **(C)** are displayed as green boxes with the white arrow inside indicating the transposase gene *tnp*. The gray-shaded areas represent areas of at least > 99% sequence identity unless otherwise indicated. This figure is modified from Kadlec et al. (2012a) and Wendlandt et al. (2015b).

The gene *spd* was initially identified in 2014 on the 3928-bp plasmid pDJ91S from a LA-MRSA ST398 of chicken origin. This plasmid was also detected in several other LA-MRSA ST398 isolates of chicken, pig, cattle, rat, and horse origins (Jamrozy et al., 2014). Plasmid pDJ91S consisted of a *rep* gene related to *repN*, a plasmid recombination gene *rec* (*pre*/*mob*) and the *spd* gene. Soon after its first description, the *spd* gene was also identified in porcine LA-MRSA ST398 and in porcine MSSA ST433 (Wendlandt et al., 2014a). In this latter strain collection, plasmid pDJ91S, but but also a slightly smaller plasmid of 3898 bp, designated pSWS2889, were present. Plasmid pSWS2889 showed the same overall structure as pDJ91S (**Table 1**). While the *spd* genes and the *rec* (*pre*/*mob*) genes of both plasmids were identical, the *rep* genes differed (**Figure 2A**). Moreover, the isolates in this latter study dated back to the year 2005, which suggested that the *spd* gene was present among LA-MRSA/MSSA isolates from animals for longer than initially thought (Wendlandt et al., 2014a).

A variant of the gene *spd* was identified on four small plasmids from porcine *S. hyicus*, *S. chromogenes*, and *S. equorum* (Wendlandt et al., 2015b). They ranged in size between 3780 and 4229 bp and had the same overall structure as pDJ91S and pSWS2889 (**Figure 2A**). However, the *rep* and *pre*/*mob* genes of all four plasmids differed from each other and from those of the two *spd*-carrying plasmids of LA-MRSA ST398. Moreover, all four novel plasmids carried a variant of the *spd* gene, which had a 12 bp deletion in the terminal part of the gene. This deletion, however, had no impact on the high spectinomycin MIC conferred by the corresponding Spd variant (Wendlandt et al., 2015b).

## Small Plasmids Carrying *apmA* Genes

The gene *apmA* is the first and so far only apramycin resistance gene described in staphylococci. This gene codes for an acetyltransferase, which is only distantly related to other acetyltransferases. It confers resistance to the aminocyclitol apramycin and also elevates the MIC values for gentamicin. The *apmA* gene was initially found on plasmid pAFS11 from a bovine LA-MRSA ST398-t2576-dt11a isolate (Feßler et al., 2011). In that study, the gene was also detected in one bovine and four porcine MRSA ST398-t011-dt11a isolates. Plasmid pAFS11 has recently been completely sequenced (Feßler et al., 2017). Its size is 49189 bp and it has a small plasmid, that harbors the genes *aadD* for kanamycin/neomycin resistance, *tet*(L) for tetracycline resistance, and *dfrK* for trimethoprim resistance, but also a *repU* gene and a *pre*/*mob* gene, integrated via IS*431* into the pAFS11 backbone (Feßler et al., 2017).

The results of another study revealed that the gene *apmA* can also reside on small plasmids. One such plasmid, the 4809-bp plasmid pKKS49, was identified in a LA-MRSA ST398 isolate that originated from a dust sample taken in a pig farm in Portugal (Kadlec et al., 2012a; **Table 1**). This plasmid had a simple composition consisting of a plasmid replication gene *rep*, three in part overlapping mobilization genes *mobA*, *mobB*, and *mobC*, and the *apmA* gene (**Figure 2B**). The corresponding Rep and Mob proteins were only distantly related to Rep and Mob proteins of staphylococci. The pKKS49-associated ApmA protein differed in 12/274 amino acids from the original ApmA protein. Based on the high apramycin MIC of 64 mg/L seen in *S. aureus* RN4220 carrying pKKS49, these amino acid substitutions seem to have no impact on the activity of the ApmA protein. Homology between both plasmids, pKKS49 and pAFS11, included only the *apmA* gene and 64 bp in the *apmA* downstream region as well as 72 bp upstream of *apmA* (Kadlec et al., 2012a).

## Small Plasmids Carrying *dfrK* Genes

The trimethoprim resistance gene *dfrK* was first described on the ca. 40-kb plasmid pKKS2187 from a porcine LA-MRSA ST398 isolate (Kadlec and Schwarz, 2009a). A closer look at the genetic environment of the *dfrK* gene revealed the apparent presence of a small plasmid consisting of a *repU* gene, a *pre*/*mob* gene, the tetracycline resistance gene *tet*(L) and the *dfrK* gene. This small plasmid was integrated via IS*257* elements into the pKKS2187 backbone. Further screening of porcine LA-MRSA ST398 isolates from Germany identified the 6243-bp plasmid pKKS627 (Kadlec and Schwarz, unpublished), which is likely to represent the progenitor plasmid of the one integrated in plasmid pKKS2187 (**Figure 2C** and **Table 1**). The *dfrK* gene has been detected not only as part of diverse larger multiresistance plasmids among MRSA ST398 isolates (Kadlec et al., 2009; Kadlec and Schwarz, 2010a; Feßler et al., 2017), but also as part of the 4289-bp non-conjugative transposon Tn*559* (Kadlec and Schwarz, 2010b; López et al., 2012).

In addition to plasmid pKKS627, the *dfrK* gene was also detected on a structurally different small staphylococcal plasmid. This plasmid, pKKS966, had a size of 4957 bp and was found in a *S. hyicus* isolate from a sow (Kadlec et al., 2012a) (**Figure 2B**). It was the first description of the *dfrK* gene in a staphylococcal species other than *S. aureus*. The plasmid, however, carried – besides *dfrK* – three *mob* genes *mobA*, *mobB*, and *mobC* as well as a *rep* gene and thus, resembled the unusual *apmA*-carrying plasmid pKKS49 (Kadlec et al., 2012a) (**Figure 2B**).

## Concluding Remarks

The data presented in this review showed that small antimicrobial resistance plasmids play a role in the dissemination of certain antimicrobial resistance genes. The observation that small plasmids similar or even identical to the ones found in LA-MRSA ST398 are present in other staphylococci, including CoNS, underlines the role of LA-MRSA ST398 as donor and/or recipient of such plasmids. Moreover, the finding that the same small plasmids occur in isolates of different geographic regions, e.g., plasmid pLNU1 in bovine CoNS from Germany and in porcine LA-MRSA ST398 and porcine *S. sciuri* from Spain, confirms that these plasmids are disseminated across animal species, bacterial species, and geographic boundaries. The observation that small staphylococcal resistance plasmids can integrate or be integrated via insertion sequences into larger plasmids or the chromosomal DNA renders them highly versatile mobile genetic elements and underlines their important role in the dissemination of antimicrobial resistance.

## Author Contributions

AF and SS wrote the first draft. All other authors provided additions and corrections. All authors listed have approved the manuscript for publication.

## Conflict of Interest Statement

The authors declare that the research was conducted in the absence of any commercial or financial relationships that could be construed as a potential conflict of interest.
